# Late-infantile GM1 gangliosidosis: A case report

**DOI:** 10.1097/MD.0000000000028435

**Published:** 2022-01-07

**Authors:** Eu Seon Noh, Hye Mi Park, Min Sun Kim, Hyung-Doo Park, Sung Yoon Cho, Dong-Kyu Jin

**Affiliations:** a Departments of Pediatrics, Samsung Medical Center, Sungkyunkwan University School of Medicine, Seoul, Korea; b Departments of Laboratory Medicine and Genetics, Samsung Medical Center, Sungkyunkwan University School of Medicine, Seoul, Korea.

**Keywords:** β-galactosidase, GLB1, GM1 gangliosidosis, joint contracture, multiple joint deformity

## Abstract

**Rationale:**

Monosialotetrahexosylganglioside (GM1) gangliosidosis is a rare lysosomal storage disorder caused by the deficiency of ß-galactosidase. Because clinical symptoms of GM1 gangliosidosis overlap with other neurodevelopmental disorders, the diagnosis of this disease is not easy, specifically in late infantile GM1 gangliosidosis. This report described a case of late-infantile GM1 gangliosidosis mistaken for juvenile idiopathic arthritis.

**Patient concerns:**

A 16-year-old girl was referred to our hospital due to persistent multiple joint deformities and mental retardation, which could not be explained by juvenile idiopathic arthritis.

**Diagnosis:**

We made a diagnosis of late infantile GM1 gangliosidosis through enzyme assays and genetic testing after a skeletal survey.

**Interventions:**

The patient underwent cervical domeplasty and laminectomy for cord compression and received rehabilitation treatment.

**Outcomes:**

The patient is receiving multidisciplinary care at a tertiary center for variable skeletal disease and conditions associated with GM1 gangliosidosis.

**Lessons:**

Late infantile GM1 gangliosidosis should be considered in the differential diagnosis of progressive neurologic decline and skeletal dysostosis.

## 1 Introduction

Monosialotetrahexosylganglioside (GM1) gangliosidosis is an autosomal recessive disorder caused by the lack of ß-galactosidase encoded by GLB1.^[1]^ It is a progressive neurodegenerative disorder, and its manifestations are caused by the accumulation of GM1 ganglioside in the central nervous system.^[2]^ GM1 gangliosidosis is classified into infantile form, late infantile form/juvenile form, and adult form according to the age of symptom onset and severity.

The infantile form is the most common and usually presents with developmental delay, seizures, and cherry red spots during the neonatal period. Patients with the late infantile form develop normally until 1 year of age, and then, they show developmental regression at 12 to 18 months of age. They have motor abnormalities and progressive diffuse brain atrophy on brain imaging. The juvenile form presents between 3 and 5 years of age. Their major symptoms are motor regression and language regression. The adult form is a slowly progressive disease, and those patients show spasticity, ataxia, dysarthria, and gradual loss of cognitive function.^[2,3]^

GM1 gangliosidosis is a rare disease. In addition, poor awareness of the disease and nonspecific clinical symptoms of GM1 gangliosidosis make diagnosis difficult and delayed.^[2]^ In this study, we report a 28-year-old Korean woman who was mistakenly diagnosed with juvenile idiopathic arthritis (JIA) during childhood and was finally diagnosed with late infantile GM1 gangliosidosis at the age of 16 years.

## 2 Case report

The female patient was born at the gestational age of 35 weeks (birth weight 3400 g; >90th percentile) by cesarean section due to bleeding from the anterior placenta. There were no perinatal problems and no specific family history. She was able to walk alone and pick up objects with her thumb and index finger until 12 months of age.

At the age of 12 months, she presented with fever, pain, and swelling of both hands. Since then, she has experienced intermittent swelling and pain on elbows, knees, shoulders, and hips, and the spine. She was hospitalized at 24 months due to the same symptoms. According to the statement of her parents, she was diagnosed with JIA at that time. She had been repeatedly hospitalized due to the same episodes until 5 years of age. After the age of 5 years, the arthritis-like symptoms disappeared; however, multiple joint contractures progressed. At the age of 12 years, she underwent quadriceps release patella tendon medial reefing due to knee joint pain, inability to stretch the legs, and bilateral dislocation of the patella. At the age of 15 years, she underwent distal femur extension osteotomy due to contracture of both knees.

**Figure 1:**
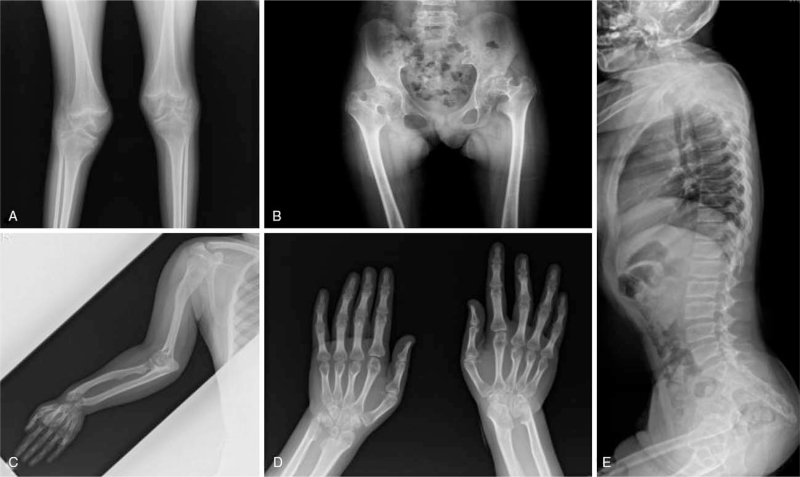
Knee and pelvic radiographs (**A, B**: patient's age:11 years old) and Right upper extremities, hands, spine lateral radiographs (**C, D, E**: patient's age: 23 years old). (**A** and **B**) Diffuse bony irregularity in articular surface of both knee. Flattened femoral head and articular surface, mild subluxation on the left superior femoral head. Secondary osteoarthritic change at both hip and knee joints. (**C**) Right upper extremity X-ray showed bowing deformities of both humerus, radius, and ulna. Both humeral heads showed deformity due to epiphyseal dysplasia. (**D**) Carpal bones and the distal epiphysis of both radius and ulna showed deformity. (**E**) Spine lateral X-ray showed congenital vertebral block in C3–C5.

**Figure 2:**
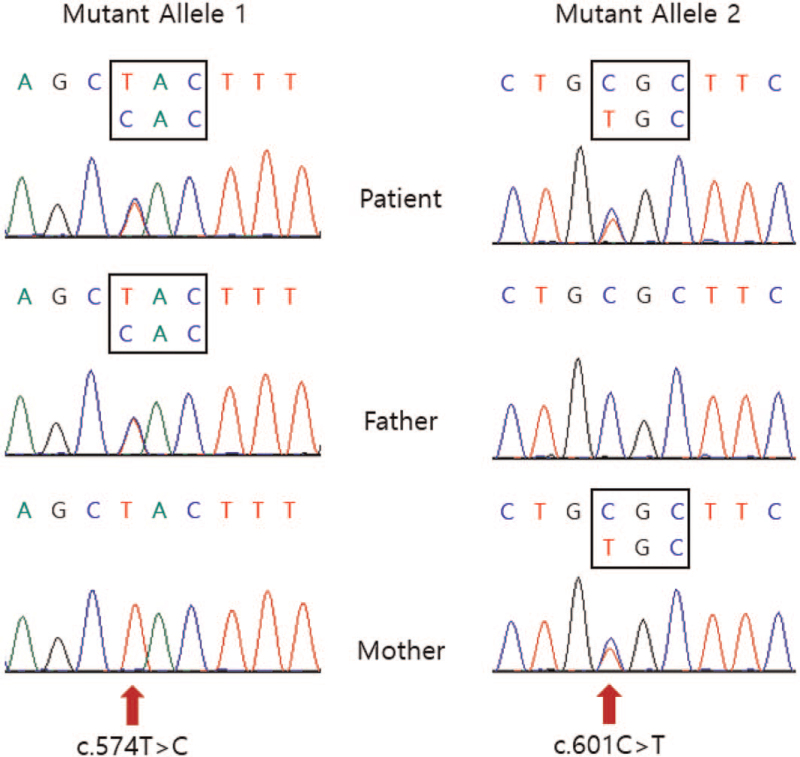
The proband had compound heterozygote mutations, c.574T>C (p.Tyr192His) and c.601C>T (p.Arg201Cys) in GLB1. Both parents were heterozygous carriers.

At the age of 16 years, she was referred to Samsung Medical Center due to persistent multiple joint deformities and mental retardation, which could not be explained by JIA. Her vital signs were stable. Her weight and height were 26 kg (−8.42 standard deviation score) and 130 cm (−5.2 standard deviation score), respectively. On physical examination, her face did not resemble that of her parents, and a prominent eye with hypertelorism and a depressed nasal bridge were noted. Both arms and fourth toes were short. She could walk when holding braces, use a spoon, and open a lid by hand. She could speak only 2 words. Blood laboratory examinations, including complete blood count, electrolyte test, liver profile, and thyroid function test, were performed, and the results were normal. Pelvic and knee X-ray in the previous hospital demonstrated degenerative changes on both hips and knees, mild subluxation on the left superior femoral head, a hypoplastic lower ileum, fossa acetabulae, a short femoral neck, squared vertebral bodies, and an irregular endplate, which were indicative of dysostosis multiplex (Fig. 1). In a urine oligosaccharide screening test, a relatively weak pattern was observed. Subsequent ß-galactosidase activity in leukocytes was 0.1 nmol/min/mg protein (reference range: 1.0–6.0 nmol/min/mg protein). Sequencing of GLB1 revealed the following 2 pathogenic variants: c.574T> C (p.Tyr192His) and c.601C> T (p. Arg201Cys). The 2 mutations were inherited from the father and mother, respectively. Finally, she was diagnosed with GM1 gangliosidosis (Fig. 2).

In the ophthalmic and dental examinations performed to evaluate GM1 gangliosidosis, corneal clouding and cherry red spots were not observed, and macroglossia and gum hypertrophy were not observed. Echocardiography findings were normal. Abdomen ultrasonography showed mild splenomegaly.

**Figure 3:**
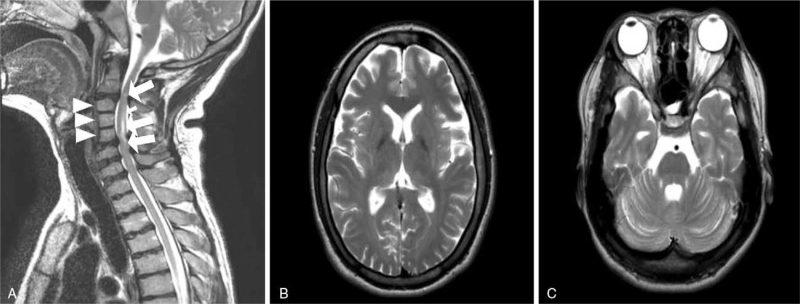
Spine MRI (**A**: patient's age: 26 years old) and brain MRI (**B, C**: patient's age: 23 years old) in the patient with the late-infantile form of GM1 gangliosidosis. (**A**) T2 sagittal image showed compressive myelopathy at the level of C2–C3 and C3–C5 (arrows). Congenital vertebral block at the level of C3–C5 and T–T2 (arrow heads) were noted. (**B**) T2 axial image showed prominent ventricles and CSF space. (**C**) T2 axial image showed diffuse atrophy of the cerebral and cerebellar hemispheres.

At the age of 26 years, a skeletal survey showed progressed secondary osteoarthritis on the overall joint area and extensive skeletal dysplasia. Spinal X-ray showed congenital block vertebrae in C3–C5, central endplate indentation and segmentation fusion anomaly, and squared vertebral bodies without odontoid hypoplasia (Fig. 1). Spinal magnetic resonance imaging (MRI) revealed complete atlanto-occipital assimilation, compressive myelopathy in C2–C3 and C3–C5, and congenital block vertebrae in C3–C5 and T1–T2. Brain MRI revealed diffuse atrophy of the cerebral hemispheres and cerebellum. Overall cerebral sulci and cerebellar folia were prominent, the extra-axial cerebrospinal fluid space was wide, and the ventricle size was slightly large (Fig. 3). At the age of 28 years, she underwent cervical domeplasty and laminectomy for cord compression and received rehabilitation treatment.

## 3 Discussion

Our patient had pain and swelling of multiple joints from 12 months to 5 years of age, and developed multiple joint deformities. She was suspected of having JIA initially. However, after 5 years of age, joint pain and swelling disappeared. The typical X-ray findings of JIA are soft-tissue swelling, joint effusion, and erosions on small and medium joints of the hand or wrist.^[4,5]^ Our patient showed X-ray findings different from those of JIA, including a hypoplastic lower ileum, fossa acetabulae, a short femoral neck, squared vertebral bodies, and an irregular endplate, which are indicative of lysosomal storage disorders.

In 1 study, which described skeletal phenotypes in patients with GM1 gangliosidosis,^[6]^ the prevalence's of odontoid hypoplasia and pear-shaped vertebral bodies were statistically higher in patients with late infantile GM1 gangliosidosis, while irregularity and central indentation of the vertebral body endplates and squared vertebral bodies were statistically more common in patients with juvenile GM1 gangliosidosis. Our patient showed central endplate indentation, an irregular endplate, and squared vertebral bodies without odontoid hypoplasia. However, pelvic X-ray showed a hypoplastic lower ilium, a hypoplastic fossa acetabula, and a subluxated femoral head. Spinal X-ray findings were similar to those of the juvenile form; however, pelvic X-ray findings were similar to those of the late infantile form. Our patient showed brachymetacarpia and brachymetatarsia (Fig. 1). Overall, our patient showed an atypical pattern compared to the patterns in skeletal surveys among other patients with GM1 gangliosidosis.^[6]^

As for late infantile GM1 gangliosidosis, there have been a few reports about an epileptic phenotype with abnormal electroencephalogram findings^[7]^ and neuroradiographic findings.^[8–11]^ Recently, 8 Korean patients with late infantile GM1 gangliosidosis were reported. In that paper, it was noted that hypomyelination on brain MRI, elevated aspartate transaminase, and abnormalities on skeletal survey were important clues for the diagnosis of GM1 gangliosidosis.^[5]^ Our patient never had seizures, and her electroencephalogram findings and aspartate transaminase levels were normal. Mild diffuse brain atrophy and mild diffuse hypomyelination were observed on brain MRI, but there was no hypointensity in the basal ganglia/globus pallidus (Fig. 3).

Until recently, 248 disease-causing mutations have been identified according to human gene mutation database (version 2020.01) in GM1 gangliosidosis. The most common mutations are missense/nonsense (74.1%), followed by small deletion (9.2%) and splicing substitutions (7.6%). The hotspot regions for GLB1 mutations appear to cluster in exons 2, 6, and 15.^[12]^ Korean patients with GM1 gangliosidosis have the most common mutation of p.D448 V in exon 13.5 However, our patient had 2 known mutations [c.601C> T (p.Arg201Cys) and c.574T> C (p.Tyr192His)] in exon 7 of GLB1. The p.Tyr192His mutation was reported previously in juvenile GM1 gangliosidosis in East Asian patients, and the p.Arg201Cys mutation was reported previously in late infantile GM1 gangliosidosis in European and Latino patients. The genotype-phenotype correlation is not definite.^[13]^

There is currently no effective treatment for GM1 gangliosidosis, and a few clinical trials are ongoing. Miglustat treatment for patients with GM1 gangliosidosis^[14]^ and combination therapy using miglustat and a ketogenic diet are currently ongoing in phase 2.^[15]^ In addition, 2 kinds of gene therapies (a single dose gene transfer vector AAV9/GLB1 or PBGM01 to deliver a functional copy of the GLB1 gene to the brain and peripheral tissues) for patients with GM1 gangliosidosis are ongoing in phase 2.^[16]^ In addition, as a treatment is currently being developed, early diagnosis is important in GM1 gangliosidosis.

In conclusion, this report describes the history from childhood to adulthood of a Korean woman diagnosed with late infantile GM1 gangliosidosis at the age of 16 years. Our patient was mistakenly diagnosed with JIA in childhood; however, she was eventually diagnosed with GM1 gangliosidosis through enzyme assays, which were performed based on developmental regression and skeletal survey findings. It is challenging to discriminate GM1 gangliosidosis from other neurodegenerative disorders, specifically in cases of the late infantile/juvenile form. Late infantile GM1 gangliosidosis should be considered in the differential diagnosis of progressive neurologic decline and skeletal dysostosis.

## Author contributions

**Conceptualization:** Euseon Noh, Hye Mi Park, Min Sun Kim, Sung Yoon Cho.

**Investigation:** Euseon Noh, Hye Mi Park, Min Sun Kim, Hyung-Doo Park, Sung Yoon Cho, Dong-Kyu Jin.

**Methodology:** Hyung-Doo Park.

**Writing – original draft:** Euseon Noh, Hye Mi Park.

**Writing – review & editing:** Euseon Noh, Hye Mi Park, Dong-Kyu Jin, Sung Yoon Cho.
